# Quality of life of children and their caregivers during an AOM episode: development and use of a telephone questionnaire

**DOI:** 10.1186/1477-7525-8-75

**Published:** 2010-07-26

**Authors:** Eve Dubé, Philippe De Wals, Manale Ouakki

**Affiliations:** 1 Quebec National Institute of Public Health, (D'Estimauville), Quebec City, (G1E 7G9), Canada; 2 Public Health Research Unit, CHUQ, (D'Estimauville), Quebec City, (G1E 7G9), Canada; 3 Department of Social and Preventive Medicine, Laval University, (avenue de la médecine) Quebec City, (G1V 0A6), Canada

## Abstract

**Background:**

The negative consequences of acute otitis media (AOM) on the quality of life (QOL) of children and their families need to be measured to assess benefits of preventive interventions.

**Methods:**

A new questionnaire was specifically designed for use in telephone surveys. A random sample of Canadian families was selected using random-digit dialling. Caregivers of children 6-59 months of age who experienced at least one AOM episode during the last 12 months were interviewed. Multidimensional severity and global QOL scores were measured both for affected children and their caregivers. Internal consistency of scores was assessed using standard tests.

**Results:**

Of the 502 eligible caregivers who completed the survey, 161 (32%) reported at least one AOM episode during the last 12 months and these cases were included in the analysis. Average severity was 2.6 for children and 2.4 for caregivers on a 1 to 4 scale (maximum severity). Cronbach alpha values were 0.78 and 0.81 for the severity score of children and caregivers respectively. Average QOL was 3.4 for children and 3.5 for caregivers on a 1 to 5 scale (best QOL). There was moderate to high correlation between severity and QOL scores, and between these scores and duration of AOM episodes.

**Conclusions:**

The questionnaire was easy to use during telephone interviews and results suggest good reliability and validity of the different scores to measure AOM severity and QOL of children and their caregivers during an AOM episode.

## Introduction

Acute otitis media (AOM) is one of the most common diseases of childhood and a leading cause of healthcare visits and antibiotic prescriptions [1]. Recurrent AOM is frequent and ≥ 3 episodes by one year of age have been reported in 10 to 19% of children [2]. In average, a child will experience four AOM episodes during the first 6 years of life [3]. AOM also disrupts daily activities of caregivers and negatively affects the lives of all household members [4,5]. Quality of life (QOL) has recently become accepted as a standard for overall policy evaluation of interventions [6]. QOL as a global and multidimensional concept, incorporates aspects of physical, functional, psychological, social, and economic well-being [7]. In the context of health care, QOL is a subjective outcome that reflects the patient's perception of his or her health status [8]. Because it is impossible to directly assess the feelings of young children, parental reports are used as a surrogate measure of their child's QOL [9]. Few instruments have been specifically designed to assess the impact of AOM on the QOL of children and their caregivers. Those available were used in face-to-face or postal surveys regarding recurrent otitis media or surgical interventions for chronic conditions [10-14]. Measurement of the severity of all AOM episodes and QOL consequences through telephone surveys is needed to assess the benefits of preventive interventions, including immunization programs against viral and bacterial infections. In the context where large numbers are needed to detect small effects in treatment and prevention and the burden to participants has to be kept as low as possible to minimise attrition bias, telephone survey is the most appropriate and cost-effective method. In 2008, 8% of Canadian households reported having cell phones only and less than 1% did not had any phone services [15]. In addition, most parents have some knowledge on AOM, a condition that could be described using a limited number of questions [16,17]. The present project reports on the development and used of a telephone questionnaire designed to measure the severity of AOM and its consequences on the QOL of the child and of the caregiver and its use in a country-wide survey in Canada.

## Methods

### Setting and study population

In May-June 2008, a telephone survey was conducted in a stratified sample of households in all Canadian provinces by a contracted company using random-digit dialling. English- or French-speaking parents or main caregivers of children 6-59 months of age were invited to participate. Questions were asked regarding the occurrence of AOM using a standard definition. The latest AOM episode in the household was selected for assessing the severity of the disease and its consequences on the QOL of the child and of the caregiver. Participation was voluntary and no incentives were given. The study protocol was approved by the Research Ethics Board of Quebec University Hospital Center (approval number 117.05.07).

### Survey instrument

Based upon the OM-6 and the Family Functioning questionnaires, a new instrument was developed for use in telephone surveys (available on request from authors). Items used for AOM severity and QOL scores are shown in Table 1.

**Table 1:** Items used to measure impact of AOM episode on the QOL of children and their caregivers during last AOM episode

Children's domains	Items
Physical suffering	Physical pain, for example pain and discomfort in the ear, fluid leaking from the ear, fever, etc. Would you say this was a <*> problem for your child?
Hearing loss	A reduction in hearing, for example, difficulty hearing, having to repeat questions you would ask him/her, the child would often ask "what", playing the TV very loud. Would you say that this was a <*> problem for your child?
Sleeping	Lack of sleep, difficulty waking up, etc. Would you say this was a <*> problem for your child?
Emotional distress	Emotional distress, for example irritability, sadness, restlessness. Would you say this was <*> problem for your child?
Activity limitations	Limitations in his/her activities, for example, playing less, doing fewer things with friends/family, not going to school or daycare, etc. Would you say this was a <*> problem for your child?
Appetite	Loss of appetite or nausea. Would you say this was a <*> problem for your child?
Children Overall QOL	How would you rate your child's quality of life during the last case of ear infection? <**>
**Caregivers' domains**	**Items**
Sleeping	Sleep difficulties, such as lack of sleep or difficulty waking up? Would you say this was a <*> problem for you?
Changing daily activities	Changes in daily activities such as housework, shopping, time spent with other children, etc. Would you say this was a <*> problem for you?
Cancelling of family activities	Cancelling family activities such as trips, vacations, outings, etc. Would you say this was a <*> problem for you?
Caregiver emotional distress	Emotional distress, such as, for example, feeling anger, irritability, frustration or sadness. Would you say this was a <*> problem for you?
Caregiver concerns	Concerns, for example, feeling worried, anxious or powerless. Would you say this was a <*> problem for you?
Overall QOL	How would you rate your quality of life during your child's last case of ear infection? <**>

*very significant, significant, not very significant, not at all significant

**very good, good, average, poor, very poor

The OM-6 is a disease-specific self-administered questionnaire covering 6 domains (physical suffering, hearing loss, speech impairment, emotional distress, activity limitations, and caregivers concerns), each one being assessed by a single question [8,18]. Two domains of the original OM-6 questionnaire were modified. The question on speech impairment, which is mainly related to recurrent AOM or otitis media with effusion and the question on the caregiver concerns were deleted. Instead, questions on sleeping disorder and on loss of appetite were added. In the original OM-6 questionnaire, answers are given on a 7-point categorical scale, and this was changed to a 4-point scale better suited to telephone interviews [19]. Scores increasing from 1 to 4 represent a problem of increasing intensity and a severity score was calculated as the mean of the scores in the six domains. The OM-6 also contains a visual analog scale of happy and sad faces allowing the caregiver to rate their child QOL on a 10-point scale. This was replaced by a question on overall QOL of the child during the last AOM episode and responses were sought on a 5-point Likert scale ranging from 1 (very poor QOL) to 5 (very good QOL).

The Family Functioning Questionnaire was developed to specifically assess the impact of recurrent AOM on the QOL of parents and families [10,11]. Four domains of the caregiver's life are covered (sleep deprivation, change of daily and social activities, emotional distress, cancelling family plans and trips), as well as two domains assessing adverse consequences for the siblings (feeling neglected and demanding extra attention). Responses are given on a 4-point Likert scale. Five domains pertaining to the caregiver were retained for the new instrument. A mean severity score was calculated representing the perceived consequences of the child's last AOM episode for caregivers. Caregiver overall QOL during last AOM episode was also assessed, using a 5-point Likert scale ranging from 1 (very poor QOL) to 5 (very good QOL).

Standard demographic variables were collected and respondents provided a description of the last episode of AOM experienced by the child, including questions on symptoms, duration of disease, complications, as well as health service use and treatment. The survey instrument was pre-tested with 10 respondents and questions requiring clarification were rewritten.

### Statistical Analyses

Descriptive statistics were generated for all variables using SAS 9.1 software. Comparisons of categorical responses were performed using chi-square or Fisher's exact tests. Mean scores were compared using the Wilcoxon rank test. Internal consistency of scores was measured by Cronbach's alpha. Inter-item correlations were calculated to reveal any redundancy in measured items and corrected item-total correlations (sum of the all item scores without including the item in question) were calculated to reveal any item that could possibly belong to a different construct than the one targeted. Correlations between severity and QOL scores and AOM duration were calculated to assess construct validity. Correlations were performed using the non-parametric Spearman test.

## Results

Of the 28,374 telephone numbers randomly generated, 26,385 were reached: 12,269 were non-residential or not in service and 8,769 were non-eligible households. In 4,796 cases, the respondent refused to participate in the survey or to answer any questions. Five hundred and fifty-one caregivers agreed to participate and 502 completed the survey, 161 of which (32%) reported at least one AOM episode in a child during the last 12 months. Characteristics of participants reporting at least one AOM episode are shown in Table 2, along with characteristics of the index child and AOM episode. Mean AOM duration was 5.9 days (median = 4 days). Twenty-seven percent of participants reported ≥3 AOM episodes in the index child during the last 12 months.

**Table 2:** Respondents' characteristics, children' characteristics and description of last AOM episode (N = 161)

Respondents' characteristics	Category	Frequency (%)	Mean, median & range
**Age, yr**	18-24	7 (4)	
	25-34	93 (58)	
	≥ 35	61 (38)	
**Link to the child**	Mother	132 (82)	
	Father	24 (15)	
	Other caregiver	5 (3)	
**Educational level**	High school diploma or less	57 (35)	
	College or university degree	104 (65)	
**Child' characteristics**			
**Age, months**	< 18	29 (18)	Mean = 37,4
	18 - < 36	43 (27)	Median = 34,3
	36 - < 54	30 (19)	Range = 5.1 -76
	≥ 54	34 (21)	
	Unknown	25 (16)	
**Gender**	Female	81 (50)	
	Male	80 (50)	
**Living in shared custody**	Yes	59 (37)	
	No	102 (63)	
**Received at least one vaccine**	Yes	159 (99)	
	No	2 (1)	
**No of AOM episodes in the past 12 mo**	1	84 (53)	Mean = 2.2
	2	32 (20)	Median = 1
	≥ 3	42 (27)	Range = 1 -10
	Unknown	3 (2)	
**Last AOM episode description**			
**Duration of the disease (days)**	≤ 3	52 (32)	Mean = 5,9
	4-6	55 (34)	Median = 4
	≥ 7	51 (32)	Range = 0-36
	Unknown	3 (2)	
**AOM symptoms reported**	Pain in the ear	139 (86)	Mean = 2.07
	Fever	130 (81)	Median = 2
	Otorrhoea/ruptured eardrum	38 (8)	Range = 0-6
	Dizziness, vertigo	23 (14)	
	Ear blocked, hearing loss	31 (19)	
	Others	53 (33)	
**Visit to a physician**	Yes	151 (94)	
	No	10 (6)	
**Caregiver absenteeism from work or school**	Yes	61 (37)	
	No	98 (61)	
	Unknown	2 (1)	

Average AOM severity scores were 2.6/4.0 for children and 2.4/4.0 for caregivers (Table 3). In children severity scores, respectively 5 and 2 respondents chose the minimal score (1 out of 4) or the maximal score (4 out of 4) for all six items and respectively 8 and 5 respondents did the same for all five items included in the caregiver severity score. Hearing loss was the only question with missing values, which was mostly observed for young children less than 3 year old. Physical suffering and sleeping disturbances were the two conditions having the highest severity scores for children. For caregivers, sleeping disturbance was the most enduring consequence of AOM.

**Table 3:** Distribution of severity scores for children and caregivers

	Not at all significant		Not very significant		Significant		Very significant		Unknown		Mean score
	(Weight = 1)		(Weight = 2)		(Weight = 3)		(Weight = 4)		(Weight = 0)		
	N	(%)	N	(%)	N	(%)	N	(%)	N	(%)	(Sd)
***Children severity score***											
Physical suffering	6	(4)	34	(21)	77	(48)	44	(27)	0	(0)	2.9 (0.8)
Hearing loss	73	(45)	49	(30)	12	(8)	14	(9)	13	(8)	1.8 (0.9)
Sleeping	18	(11)	40	(25)	56	(35)	47	(29)	0	(0)	2.8 (0.9)
Emotional distress	17	(11)	39	(24)	65	(40)	40	(25)	0	(0)	2.8 (0.9)
Activity limitations	21	(13)	35	(22)	68	(42)	37	(23)	0	(0)	2.7 (0.9)
Appetite	22	(14)	55	(34)	48	(30)	36	(22)	0	(0)	2.6 (0.9)
Severity score (mean)											2.6 (0.7)
***Caregivers severity score***											
Sleeping	25	(16)	40	(25)	51	(32)	44	(27)	1	(0)	2.7 (1.0)
Changing daily activities	30	(19)	51	(32)	53	(33)	26	(16)	1	(0)	2.5 (0.9)
Cancelling of family activities	66	(41)	56	(35)	25	(16)	14	(9)	0	(0)	1.9 (0.9)
Caregiver emotional distress	55	(34)	57	(35)	31	(19)	18	(11)	0	(0)	2.1 (1.0)
Caregiver concerns	24	(15)	40	(25)	71	(44)	26	(16)	0	(0)	2.6 (0.9)
Severity score (mean)											2.4 (0.7)

The distribution of QOL scores for children and caregivers is shown in Table 4. The average QOL score was 3.4/5.0 for children and 3.5/5.0 for caregivers. The median mark was the most frequently reported QOL during AOM episodes, both for children and caregivers. A very poor QOL was reported in 3% of AOM cases.

**Table 4:** Distributions of QOL scores for children and caregivers

	1		2		3		4		5			
	Very poor		Poor		Average		Good		Very Good		Mean score	
	N	(%)	N	(%)	N	(%)	N	(%)	N	(%)	(Sd)	
***Children***												
Overall QOL	5	(3)	27	(17)	57	(35)	41	(25)	31	(19)	3.4 (1.1)	
***Caregivers***												
Overall QOL	5	(3)	17	(11)	63	(39)	52	(32)	24	(15)	3.5 (1.0)	

Cronbach alpha values were 0.78 and 0.81 for severity scores in children and caregivers, respectively. As shown in Table 5, correlation coefficients between the variables composing the severity scores were in the expected range and no redundancy was identified. The corrected item-total correlation coefficients did not reveal any outlier in the items. Cronbach alpha values for analyses excluding one item were always lower than the overall Cronbach coefficient value for the total score, suggesting the absence of any redundancy in measured items.

**Table 5:** Children and caregivers severity scores: Inter-Item, Item-Total Correlations and Cronbach Alpha Reliability Estimates

Children severity score	Physical suffering	Hearing loss	Sleeping	Emotional distress	Activity limitations	Appetite	Corrected item-total correlation	Cronbach's Alpha if Item Deleted
Physical suffering	1,00	0,33	0,34	0,34	0,47	0,37	0,54	0,74
Hearing loss		1,00	0,22	0,23	0,31	0,33	0,39	0,77
Sleeping			1,00	0,38	0,33	0,34	0,45	0,76
Emotional distress				1,00	0,52	0,4	0,55	0,74
Activity limitations					1,00	0,56	0,64	0,71
Appetite						1,00	0,57	0,73
**Caregivers severity score**	**Sleeping**	**Changing daily activities**	**Cancelling of family activities**	**Caregiver emotional distress**	**Caregiver Concerns**		**Corrected item-total correlation**	**Cronbach's Alpha if Item Deleted**
Sleeping	1,00	0,65	0,49	0,39	0,42		0,64	0,76
Changing daily activities		1,00	0,61	0,43	0,34		0,67	0,75
Cancelling of family activities			1,00	0,43	0,28		0,59	0,78
Caregiver emotional distress				1,00	0,55		0,58	0,78
Caregiver concerns					1,00		0,51	0,80

Table 6 reflects the correlations between severity and QOL scores, and between these two scores and AOM episode duration. Results indicate moderate correlation between children's severity and overall QOL scores (Spearman coefficient = 0.38) and between caregivers' severity and overall QOL scores (Spearman coefficient = 0.29). However, there was a high correlation between severity scores (Spearman coefficient = 0.69) and between QOL scores (Spearman coefficient = 0.65) for children and caregivers. Duration of AOM episode was moderately correlated with the four scores (Spearman coefficient between 0.22 and 0.13). Not shown in the Table, all scores were significantly different between children with severe AOM, (i.e. AOM that lasted ≥ 4 days and had ≥ 3 related-symptoms, n = 56) and children with less severe AOM, (i.e. AOM that lasted ≤ 3 days and had ≤ 2 related-symptoms, n = 105) (p < 0.0001).

**Table 6:** Spearman's correlations between severity scores, QOL scores and duration of AOM episode

Item	Children severity score	Caregivers severity score	Children QOL score	Caregivers QOL score	Duration of AOM episode
Children severity score	1,00	0,69*	0,38*	**0,29**^**†**^	**0,16**^**¥**^
Caregivers severity score		1,00	0,31*	0,45*	**0,22**^**†**^
Children overall QOL			1,00	0,65*	**0,21**^**†**^
Caregivers overall QOL				1,00	0,13
Duration of AOM episode					1,00

*P < 0.0001; ^† ^P < 0.01; ^¥ ^P < 0.05

## Discussion

The questionnaire tested in the present survey was specifically designed for telephone interviews, the most practical method to estimate the social burden of disease in the North American context [19,20]. This newly developed instrument focuses on the adverse consequences of AOM both for children and their caregivers. The inclusion of questions pertaining to sleeping disturbances was a useful addition, as this specific problem is frequent and important during AOM episodes, both for children and their caregivers.

The percentage of missing values was minimal which underlines the feasibility of telephone interviews. The questionnaire demonstrated minimal floor and ceiling effects with no more than 5% of respondents having minimum (floor effect) or maximum (ceiling effect) scores for all scores. Identification of changes in AOM severity would be possible using this instrument. Corrected item-total correlations of all items included in the two severity scores were above 0.30, which indicate high discrimination.

Previous studies have shown the negative impact of recurrent or chronic otitis media on parental stress, family functioning and parents' perception of children's QOL [4,5,7,8,14,21]. In economic evaluations of pneumococcal conjugate vaccine programs, QOL losses were considered for children only [22] or for both children and parents [23]. Results of the present study support the latter approach.

Our study has several limitations. Obviously, the QOL of healthy children recruited in this survey was not assessed before or after the AOM episode. However, we can reasonably assume that most of them had a very good QOL (maximum score of 5 out of 5), as supported by results of health surveys in the US [16,17,24]. Test-retest reliability of the questionnaires was not assessed. As results of this study suggest that the questionnaire is a useful tool to measure the severity of AOM and its consequences on the QOL, more detailed reliability and validity information should be obtain. The questionnaire used in this study was based upon two other instruments: OM-6 and Family Functioning questionnaires. In previous studies, test-retest reliabilities of those questionnaires were measured and results were very satisfactory [12,13]. Extending the recall period to AOM episodes occurring during the last 12-month period may have decreased accuracy in reporting, and a systematic bias could have been generated if only the more severe outcomes were reported or if the recollection of the severity of a given AOM episode is modified as time passes. One could also argue that caregivers' perceptions regarding adverse consequences of AOM may have influenced the proxy rating of the child's QOL [14]. A study suggested that the mothers of children who experienced recurrent episodes of AOM rated their children as significantly more demanding compared to healthy children. These mothers also rated themselves as significantly more depressed and less competent than did control mothers [21]. The high correlation founded between both scores for children and caregivers may indicate a projection bias from caregivers as necessary proxy respondent for their child. However, it is usually impractical to obtain judgments of QOL from young children and this limitation is inherent to most research in the QOL domain for children [17]. Finally, the fact that severity scores were only moderately correlated with overall QOL scores suggests that unmeasured psychological factors influence QOL rating of AOM by caregivers.

## Conclusion

The questionnaire developed for this study on AOM has shown good reliability and satisfactory construct validity, and is easy to use in telephone interviews. AOM has adverse consequences both for children and their caregivers and this fact should be taken into account in future studies.

## Competing interests

This study was financially supported by an unrestricted grant from GlaxoSmithKline. The sponsor was not involved in study protocol/questionnaire designing, data collection or data analysis and interpretation.

## Authors' contributions

All authors have been involved in the design of the study. ED and PDW have drafted the manuscript. MO performed the statistical analysis. All authors have read and approved the final version of the manuscript.

## References

[B1] O Klein J The burden of otitis media Vaccine 2001 19 S1 S2 S8 10.1016/s0264-410x(00)00271-1 11163456

[B2] Casselbrant ML Mandel EM Rosenfeld RM, Bluestone CD Epidemiology Evidence-based otitis media 1999 Ontario: Decker 117 136

[B3] Giebink GS Rosenfeld RM, Bluestone CD Prevention Evidence-based otitis media 1999 Ontario: Decker 223 234

[B4] Asmussen L Olson L Sullivan SA «You have to live it to understand it ...» - Family Experiences with chronic otitis media in children Ambulatory Child Health 1990 5 303 312

[B5] Boruk M Lee P Faynzilbert Y Rosenfeld RM Caregiver well-being and child quality of life Otolaryngology - Head and Neck Surgery 2007 146 159 168 10.1016/j.otohns.2006.09.005 17275533

[B6] Drummond MF Methods for the economic evaluation of healthcare programmes 1997 Second Toronto: Oxford Medical Publications 305

[B7] Haggard MP Smith SC Rosenfeld RM, Bluestone CD Impact of otitis media on child quality of life Evidence-based otitis media 1999 Ontario: Decker 375 398

[B8] Rosenfeld RM Goldsmith AJ Tetlus L Balzano A Quality of life for children with otitis media Arch otolaryngol Head Neck Surg 1997 123 1049 1054 9339979 10.1001/archotol.1997.01900100019002

[B9] Epstein AM Hall JA Tognetti J Son LH Conant L Using proxies to evaluate quality of life Med Care 1989 27 Suppl 3 S99 S108 2921889

[B10] Brouwer CN Effect of pneumococcal vaccination on quality of life in children with recurrent acute otitis media: a randomized, controlled trial Pediatrics 2005 115 2 273 9 10.1542/peds.2004-0778 15687432

[B11] Brouwer CNM Reliability and validity of functional health status and health-related quality of life questionnaires in children with recurrent acute otitis media Qual Life Res 2007 16 1357 1373 10.1007/s11136-007-9242-0 17668290 PMC2039822

[B12] Rosenfeld RM Quality of life for children with otitis media Arch otolaryngol Head Neck Surg 1997 123 1049 1054 9339979 10.1001/archotol.1997.01900100019002

[B13] Boruk M Caregiver well-being and child quality of life Otolaryngology - Head and Neck Surgery 2007 146 159 168 10.1016/j.otohns.2006.09.005 17275533

[B14] Brouwer CNM Rovers MM Maillé AR Veenhoven RH Grobbee DE Sanders EAM Schilder AGM The impact of recurrent acute otitis media on the quality of life of children and their caregivers Clinical Otolaryngology 2005 30 258 265 10.1111/j.1365-2273.2005.00995.x 16111423

[B15] Statistics Canada Residential Telephone Service Survey [En ligne] 2010 http://www.statcan.gc.ca/daily-quotidien/090615/dq090615c-eng.htm [cited 2010 Page consulted April 26, 2010]

[B16] Alsarraf R Otitis media health status evaluation: a pilot study for the investigation of cost-effective outcomes of recurrent acute otitis media treatment Ann Otol Rhinol Laryngol 1998 107 2 120 8 9486906 10.1177/000348949810700207

[B17] Haggard MP Smith SC Rosenfeld RM, Bluestone CD Impact of otitis media on child quality of life Evidence-based otitis media 1999 Ontario:Decker 375 398

[B18] Kubba H Swan IRC Gatehouse S How Appropriate is the OM6 as a Discriminative Instrument in Children with Otitis Media? Arch otolaryngol Head Neck Surg 2004 130 705 709 10.1001/archotol.130.6.705 15210550

[B19] Dillman DA Mail and telephone surveys - The total desing method 1978 A Wiley-Interscience publication ed. United States: John Wiley & Sons 325

[B20] Choi CKB L'interview téléphonique assistée par ordinateur (ITAO) dans les enquêtes sur la santé à des fins de surveillance de la santé publique: questions d'ordre méthodologique et défis à relever 2004 25 2 Maladies chroniques au Canada 23 30

[B21] Forgays DK Hasazi JE Wasserman RC Recurrent otitis media and parenting stress in mothers of two year-old children Dev Behav Pediatr 1992 13 5 321 325 10.1097/00004703-199210010-00001 1401114

[B22] Melegaro A Edmunds WJ Cost-effectiveness analysis of pneumococcal conjugate vaccination in England and Wales Vaccine 2004 22 31-32 4203 14 10.1016/j.vaccine.2004.05.003 15474710

[B23] Poirier B De Wals P Petit G Erickson LJ Pépin J Cost-effectiveness of a 3-dose pneumococcal conjugate vaccine program in the province of Quebec, Canada Vaccine 2009 7105 9 10.1016/j.vaccine.2009.09.057 19786137

[B24] Erickson P Wilson R Shannon I Years of healthy life Healthy People 2000 Stat Notes 1995 7 1 15 11762385 10.1037/e583992012-001

